# Genome and secretome analysis of the hemibiotrophic fungal pathogen, *Moniliophthora roreri*, which causes frosty pod rot disease of cacao: mechanisms of the biotrophic and necrotrophic phases

**DOI:** 10.1186/1471-2164-15-164

**Published:** 2014-02-27

**Authors:** Lyndel W Meinhardt, Gustavo Gilson Lacerda Costa, Daniela PT Thomazella, Paulo José PL Teixeira, Marcelo Falsarella Carazzolle, Stephan C Schuster, John E Carlson, Mark J Guiltinan, Piotr Mieczkowski, Andrew Farmer, Thiruvarangan Ramaraj, Jayne Crozier, Robert E Davis, Jonathan Shao, Rachel L Melnick, Gonçalo AG Pereira, Bryan A Bailey

**Affiliations:** 1 Sustainable Perennial Crops Lab, USDA/ARS, Bldg 001 Rm 223 Beltsville Agricultural Research Center-West, Beltsville, MD 20705, USA; 2 Centro Nacional de Processamento de Alto Desempenho em São Paulo, Universidade Estadual de Campinas, CP 6141, Campinas 13083-970, SP, Brazil; 3 Laboratório de Genômica e Expressão, Departamento de Genética, Evolução e Bioagentes, Instituto de Biologia, Universidade Estadual de Campinas (UNICAMP), CP 6109, Campinas 13083-970, SP, Brazil; 4 Center for Infectious Disease Dynamics, Pennsylvania State University, University Park, PA 16802, USA; 5 Department of Ecosystem Science and Management, Pennsylvania State University, University Park, PA 16802, USA; 6 Department of Horticulture, Pennsylvania State University, University Park, PA 16802, USA; 7 Department of Genetics, School of Medicine, University of North Carolina at Chapel Hill, Mary Ellen Jones, Room 921, 27599-3280 Chapel Hill, NC, USA; 8 National Center of Genomic Research, 2935 Rodeo Park Drive East Santa Fe, NM 87505 Santa Fe, USA; 9 CABI Bioscience UK Centre, Egham, UK; 10 Molecular Plant Pathology Lab, USDA/ARS, Bldg 004 Rm 119 Beltsville Agricultural Research Center West, Beltsville, MD 20705, USA

## Abstract

**Background:**

The basidiomycete *Moniliophthora roreri* is the causal agent of Frosty pod rot (FPR) disease of cacao (*Theobroma cacao*), the source of chocolate, and FPR is one of the most destructive diseases of this important perennial crop in the Americas. This hemibiotroph infects only cacao pods and has an extended biotrophic phase lasting up to sixty days, culminating in plant necrosis and sporulation of the fungus without the formation of a basidiocarp.

**Results:**

We sequenced and assembled 52.3 Mb into 3,298 contigs that represent the *M. roreri* genome. Of the 17,920 predicted open reading frames (OFRs), 13,760 were validated by RNA-Seq. Using read count data from RNA sequencing of cacao pods at 30 and 60 days post infection, differential gene expression was estimated for the biotrophic and necrotrophic phases of this plant-pathogen interaction. The sequencing data were used to develop a genome based secretome for the infected pods. Of the 1,535 genes encoding putative secreted proteins, 1,355 were expressed in the biotrophic and necrotrophic phases. Analysis of the data revealed secretome gene expression that correlated with infection and intercellular growth in the biotrophic phase and invasive growth and plant cellular death in the necrotrophic phase.

**Conclusions:**

Genome sequencing and RNA-Seq was used to determine and validate the *Moniliophthora roreri* genome and secretome. High sequence identity between *Moniliophthora roreri* genes and *Moniliophthora perniciosa* genes supports the taxonomic relationship with *Moniliophthora perniciosa* and the relatedness of this fungus to other basidiomycetes. Analysis of RNA-Seq data from infected plant tissues revealed differentially expressed genes in the biotrophic and necrotrophic phases. The secreted protein genes that were upregulated in the biotrophic phase are primarily associated with breakdown of the intercellular matrix and modification of the fungal mycelia, possibly to mask the fungus from plant defenses. Based on the transcriptome data, the upregulated secreted proteins in the necrotrophic phase are hypothesized to be actively attacking the plant cell walls and plant cellular components resulting in necrosis. These genes are being used to develop a new understanding of how this disease interaction progresses and to identify potential targets to reduce the impact of this devastating disease.

## Background

Fungal plant pathogens can be classified as biotrophic, necrotrophic or hemibiotrophic pathogens based on how they interact with their host. Biotrophic pathogens cause only minor responses from the plant, particularly at initial stages of the disease. These biotrophic pathogens appear to evade plant defenses with stealthy methods [1]. Fungal biotrophs are often obligate pathogens, typically having narrow host ranges, possessing haustoria and secreting limited amounts of lytic enzymes [2]. On the other hand, infection by necrotrophic pathogens causes rapid cell death in hosts and elicit major molecular responses from the plant. Necrotrophs appear to utilize brute force and overwhelm the plant defenses. Necrotrophs are typically non-obligate pathogens, have wide host ranges and secrete copious amounts of lytic enzymes and toxins [2]. Hemibiotrophs initiate infection with a period of biotrophy, followed by a necrotrophic phase, and they possess properties of both groups. However, most of our understanding of how hemibiotrophs interact with their hosts is derived from these two extremes.

Both biotrophic and necrotrophic fungi share common elements but these may have different purposes when causing disease. During the host-interaction, the pathogens synthesize and secrete various peptides/proteins that block host responses (biotrophs) or kill the host cells (necrotrophs). Among biotrophs, the rust fungus of flax, *Melampsora lini,* excretes cysteine-rich avirulence elicitor proteins from the haustoria [3] and the bean rust fungus *Uromyces fabae* shows highly coordinated stage specific regulation of its secreted proteins [4]. The necrotrophic fungus *Fusarium graminearum* has 109 secreted cell wall degrading genes in its genome [5], while *Alternaria* species secrete non-host and host specific toxins that disrupt photosynthesis and kill plant cells [6]. Therefore, a detailed understanding of specific peptides/proteins secreted during the host-pathogen interaction is vital to elucidate the biotrophic and necrotrophic mechanisms.

*Moniliophthora roreri* (Cif.) H.C. Evans, Stalpers, Samson & Benny [7] causes Frosty Pod Rot (FPR), a devastating pod disease of *Theobroma cacao L.* (cacao), the source of cocoa powder and cocoa butter. Phylogenetically, *M. roreri* is related to another fungal pathogen, *Moniliophthora perniciosa* (Stahel) Aime and Phillips-Mora [8], which causes Witches’ Broom Disease (WBD), a disease that infects all cacao meristematic tissues including flowers, shoots, and pods [8]. Together, these pathogens cause two of the most economically important diseases of *Theobroma cacao* in the Western Hemisphere [9,10]. While both of these fungal species are pathogenic on the plant genera *Theobroma* and *Herrania*, *M. roreri* is not known to have any other hosts, whereas *M. perniciosa* has distinct biotypes that infect different host species [11]. Historically, these *Moniliophthora* pathogens have spread independently to cacao producing areas across the Western Hemisphere and they have typically resulted in production losses of 75% or higher in nearly all the cacao growing regions in the Americas [10,12].

*Moniliophthora roreri* and *M. perniciosa* are both hemibiotrophic pathogens, but have distinctive lifestyles and pathogenicity strategies. Unlike most other hemibiotrophic fungi, both *Moniliophthora* species have protracted biotrophic stages or phases that last three to six weeks. They also have distinctly different mycelial morphologies present at the beginning and the end of the disease process [10,13]. In both *Moniliophthora* diseases, the infected plant tissues are asymptomatic for 14 to 21 days. After that period of time, these tissues typically begin to show some form of altered growth or swelling that continues for the remainder of the biotrophic phase, culminating with the necrosis of the host tissues, which marks the beginning of the necrotrophic phase. In other hemibiotrophic fungi such as *Cladosporium fulvum, Mycosphaerella graminicola, Pyrenopeziza brassicae* and *Septoria tritici,* the biotrophic phase is asymptomatic with undifferentiated mycelia and lasts no more than two weeks [14].

As with hemibiotrophic *Colletotrichum* species, both *M. roreri* and *M. perniciosa* undergo distinct metabolic changes as they transition from the biotrophic phase to the necrotrophic phase of growth [15-19]. These metabolic changes suggest significant shifts in gene expression and in enzymatic processes.

In this study we present the genome of *M. roreri* and compare it to the genome of the closely related cacao pathogen, *M. perniciosa*. Transcriptional analysis of the genome will focus on genes with putative signal peptides from the *M. roreri* genome and compare them with expressed genes, identified through RNA sequencing of infected pods, to discover potential secreted proteins expressed during the disease process. Genes expressed in the early and late stages of FPR on pods were used to identify secreted genes that are potentially important during the biotrophic or necrotrophic phase of the disease cycle. The results presented here are the first analyses of genes encoding secreted proteins derived from the *M. roreri* genome. These new insights into the *M. roreri* genome, transcriptome and differential gene regulation provide a better understanding of the plant-pathogen interactions that occur during FPR.

## Results

### Genome structure

*Moniliophthora roreri* has a genome size of 52.3 Mbp, which is 7.7 Mbp larger than the 44.6 Mbp found in the *M. perniciosa* genome (Table 1). Despite the size difference, *M. roreri* had only 912 additional coding sequences (CDS) than *M. perniciosa.* 17,920 coding sequences were detected in M*. roreri.* The average GC% for the *M. roreri* genome (46.88%) is lower than for the *M. perniciosa* genome (47.81%); however, the average G + C for the *M. roreri* CDS is nearly identical to the value for *M. perniciosa*. Gene density for *M. roreri* was also found to be lower than for *M. perniciosa*.

**Table 1:** **Genome Comparisons of *M. roreri *** **and *M. perniciosa***

	**Genome size (bp)**	**Average (G + C)%**	**Total number contigs**	**N50 (bp)**	**Max contig size (bp)**	**Min contig size (bp)**	**Median contig size**	**Total tRNA**	**Avg gene Density**	**Total CDS**	**Avg (G + C) CDS%**	**Maximum CDS size (bp)**	**Median CDS size (bp)**
*M. roreri*	52,334,075	46.88%	3,298	48,134	571,142	367	5,455	670*	0.42185#	17,920	49.55%	15,081	1,023
*M. perniciosa*	44,661,472	47.81%	3,087	48,096	476,325	500	3,300	550	0.51058#	17,008	49.83%	15,048	1,095

*using tRNAscan default parameters yielded 331 contigs that have tRNA for a total of 670 tRNA’s.

#CDS bases/total genome bases.

An analysis of the genomes of these pathogens has revealed that retro-transposons (LTRs) contribute to about 6.6% (3.46 Mbp) of the *M. roreri* genome compared to about 0.74% (0.332 Mbp) in the *M. perniciosa* genome. In *M. perniciosa*, transposable elements were reported to be active elements that may contribute to genetic variability of this pathogen [20]. Each genome was scanned for repetitive sequences and low complexity DNA using the Repeat Modeler and RepeatMasker programs. *M. roreri* had 7,060,129 bases associated with repeats and repetitive elements while *M. perniciosa* had 1,737,865 bases associated with repeats and repetitive elements (Additional file 1).

### Synteny

**Figure 1:**
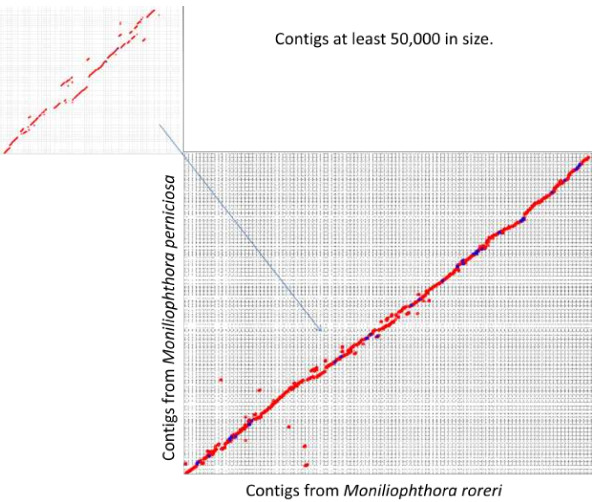
**MUMmer alignment dot plot of *M. roreri *and *M. perniciosa *contigs using only contigs 50 kb or larger.** The line graph represents the MUMmer results for the nucleotide comparison of *M. roreri* contigs to *M. perniciosa* contigs with a size cutoff of 50 kb. Only contigs larger than 50 kb were compared and contigs with no corresponding homology were eliminated from this particular analysis. Red circles represent positive strand alignments while blue circles represent negative strand alignments. The insert represent an enlargement of the MUMmer graph at that location. The 50 kb contigs utilized in this analysis for *M. roreri* account for 23,142,326 bp or 44% of the total 52,334,081 bp genome, where as for *M. perniciosa* 21,535,677 bp were, used which represents 48% of the 44,661,472 bp genome.

Using a bi-directional blast with an E-value of E-04 as the cut-off value for two proteins being considered a match, a whole genome comparison of the predicted proteins between the *M. roreri* and *M. perniciosa* genomes was performed. This analysis revealed that 16,713 protein encoding genes (93%) from *M. roreri* have sequence similarity to *M. perniciosa* protein encoding genes and 15,674 protein encoding genes (92%) from *M. perniciosa* have sequence similarity to *M. roreri* protein encoding genes. At lower E-values, E-25 and E 0.0, the *M. roreri* genome open reading frames (ORFs) are 87% and 40% similar to the *M. perniciosa* genome ORFs, respectively. In *M. roreri* there are 8,851 predicted genes or 49% that have no predicted function and are listed as hypothetical genes. Synteny between *M. roreri* and *M. perniciosa* was analyzed with the program MUMmer using the NUCmer setting that compares the contigs from both genomes at the nucleotide level. In this analysis, *M. roreri* contigs were queried using *M. perniciosa* contigs as the reference sequence. Contigs with high sequence identity resulted in a straight line graph, while translocations and inversions result in either a parallel shift in the line (up or down) or a line at a negative angle, respectively. Though both genomes are closely related at the nucleotide level, there are regions where inversions and translocations have potentially occurred. This analysis confirms that these two genomes are very similar and most probably homologous, while it also shows that some regions in both genomes are unique. Due to the poor resolution of the MUMmer plot when all genome contigs were analyzed, we compared long intact regions of the genomes. Two hundred and fifty contigs from *M. roreri* and 233 contigs from *M. perniciosa* were compared that were at least 50 kb in length. Contigs with no corresponding sequence identity were eliminated, so that only contigs of high sequence identity between the two genomes were compared (Figure 1). The results yielded a final comparison of 222 contigs from *M. roreri* and 207 contigs from *M. perniciosa* and showed highly similar sequences with slight shifts off the diagonal line, which indicate translocations in the contigs (Figure 1 insert that shows an expanded region of the main figure).

### Comparisons to other basidiomycete fungi

**Figure 2:**
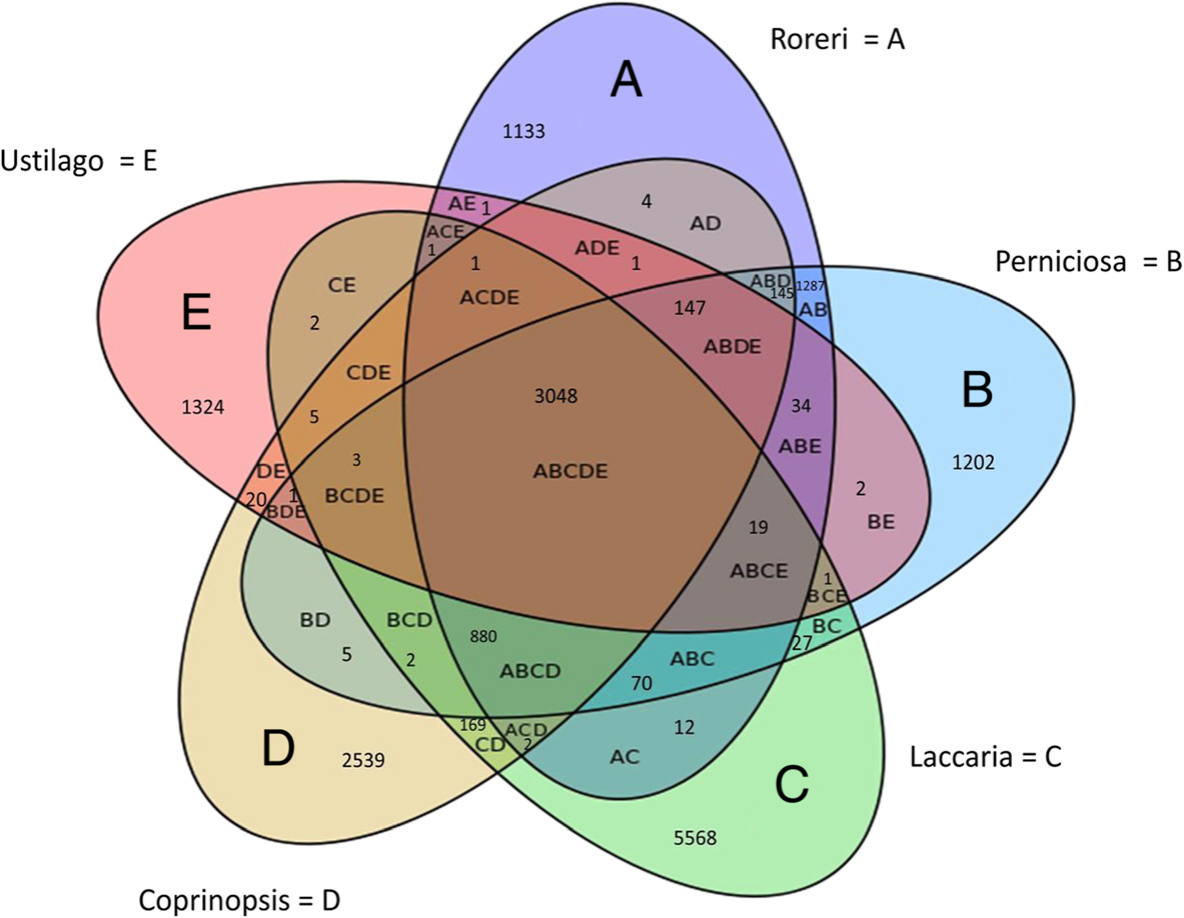
**Bi-directional Venn diagram.** Bi-directional blast results are present in a Venn diagram. The code used for this diagram is: **A** oval = *M. roreri* genes; the **B** oval = *M. perniciosa* genes; **C** oval = *L. bicolor* genes; **D** oval = *C. cinerea* genes and **E** oval = *U. maydis* genes. Intersects are labeled with the corresponding letters and a number which represents the number of specific genes in that particular intersect. An E-value E-04 was used as the homology cut off.

Bidirectional blast analysis was conducted with *M. roreri, M. perniciosa, Laccaria bicolor, Coprinopsis cinerea (Coprinus cinereus)*, and *Ustilago maydis*. These other genomes were selected because all are basidiomycetes but exhibit differing environmental adaptations representing a mushroom producing ectomycorrhizae a mushroom producing saprophyte and a pathogenic smut fungus, respectively. Figure 2 presents the Venn-diagram of the analysis, and the tax plot is shown in Table 2. Each of the five genomes is represented by an oval, and the numbers indicate how many genes are included within that intersect, at an E-value of E-04. Using this analysis, genes can be identified that are exclusive to a particular genome or to a particular group, which enables identification of putative similarities such as pathogenicity genes, and genus specific genes. The number of predicted genes particular to a single genome is: 1,133 for (A) *M. roreri*, 1,202 for (B) *M. perniciosa*, 5,568 for (C) L. bicolor, 2,539 for (D) *C. cinerea* and 1,324 for (E) *U. maydis*. Only seven of the 1,133 *M. roreri* genes were detected by RNA-Seq analysis (described below) and only 16 of the 1,133 *M. roreri* genes have putative functions (Table 3). Based on the annotation, several of these genes appear to have a role in genome integrity and function (a RNA polymerase Rpb1 C-terminal repeat domain-containing protein; a putative RecQ helicase; a P-loop containing nucleoside triphosphate hydrolase protein) while another set appears to be linked to retro elements (a Gag protein, retrovirus-related Pol polyprotein from transposon TNT 1–94 and a reverse transcriptase protein). The remaining genes have similarity to a mix of toxins (volvatoxin A2 precursor, a dipterans toxic crystal protein); heme binding (a Hemopexin domain-containing protein); cell adhesion (a calcium-binding tyrosine phosphorylation-regulated protein); pectin degradation (a probable pectin lyase precursor); possible oxidoreductases (two FAD NAD-binding domain-containing proteins); a putative CNVH-domain-containing protein and two capsule polysaccharide biosynthesis proteins. The ABCDE intersection contains 3,048 genes (data not shown), which are common to all of these basidiomycetes, while there are 34 genes associated with ABE and 70 with ABC. The *Moniliophthora* intersect (AB) has 1,287 genes, of which only 106 or 8% have a putative function (Table 4). Among the AB genes there are five antibiotic biosynthesis monooxygenases; five feruloyl esterases involved in hemicellulose degradation; four cellulose binding proteins; four cell wall glycosyl hydrolases, involved in cell wall sugar degradation; four chitin binding proteins; four het domain proteins associated with fungal vegetative incompatibility and mycelial cell death; three pectate lyases involved in pectin degradation; three family 12 carbohydrate esterases, a family that includes pectin acetylesterases, rhamnogalacturonan acetylesterases and acetyl xylan esterases; three biotrophy-associated secreted proteins that are putative effectors and 2 amidohydrolases, enzymes that act on amide bonds. Most of these AB intersect genes were constitutively expressed, but RNA-Seq data also showed differential gene expression for 14 genes, which are indicated in Table 4 (Additional file 1). In the necrotrophic phase the following genes were upregulated: an antibiotic biosynthesis monooxygenase, two biotrophy-associated secreted proteins, a cellulose binding protein, a FAD binding domain protein, an integral membrane protein, a lactam utilization protein, a LEA domain-containing protein, a thaumatin-like protein, a transferase family protein and a urea hydro-lase cyanamide protein. In the biotrophic phase a cell wall glycosyl hydrolase, a family 16 glycoside hydrolase and a NAD binding phosphogluconate dehydrogenate-like protein were upregulated (Additional file 1, AB intersect sheet). The pathogenic intersect (ABE) has 30 genes with known functions and these are shown in Table 5. The biotrophic intersect (ABC) has 17 genes with putative functions (Table 6). RNA-Seq data revealed that most of ABC and ABE intersect genes are constitutively expressed (Additional file 1) at low to mid levels (adjusted means at 2 to 50 reads = Low level; 50 to 125 reads = mid level and >125 = high level of gene expression), with the exception of two genes from the ABC intersect, (a transcriptional family alpha/beta fold family protein and one hypothetical protein) that were differentially expressed in the necrotrophic phase (Additional file 1, ABE and ABC intersect sheets).

**Table 2:** Tax plot results of the bi-directional blast analysis

**Bidirectional blast results**				**Venn intersection #**
**Number of genes with homology at an E-value E0-4**				
A-1133				1133
AB-2982	BA-2508			1287
AC-34	CA-86			12
AD-13	DA-26			4
AE-2	EA-17			1
ABC-866	BAC-526	CAB-479		70
ABD-540	BAD-486	DAB-395		145
ABE-136	BAE-125	EAB-100		34
ACD-25	CAD-101	DAC-57		2
ACE-0	CAE-2	EAC-5		1
ADE-0	DAE-2	EAD-8		1
ABCD-3650	BACD-3819	CABD-3739	DABC-2957	880
ABCE-192	BACE-144	CABE-89	EABC-123	19
ABDE-348	BADE-355	DABE-265	EABD-254	147
ACDE-0	CADE-8	DACE-6	EACD-8	1
ABCDE-7998	BACDE-7711	CABDE-6535	DABCE-6126	3048
B-1202				1202
BC-61	CB-249			27
BD-33	DB-71			5
BE-2	EB-22			2
BCD-38	CBD-366	DBC-182		2
BCE-0	CBE-5	EBC-9		1
BDE-1	DBE-2	EBD-7		1
BCDE-5	CBDE-18	DBCE-9	EBCD-22	3
C-5568				5568
CD-904	DC-659			169
CE-22	EC-40			2
CDE-44	DCE-31	ECD-37		5
D-2539				2539
DE-29	ED-53			20
E-1324				1324

**Table 3:** ***M. roreri *** **intersect**

**A intersect**	
**Gene Id**	**Gene annotation**
evm.model.sctg_0272_0002.2	Calcium-binding tyrosine phosphorylation-regulated
evm.model.sctg_0180_0002.8	Capsule polysaccharide biosynthesis
evm.model.sctg_0180_0002.6	Capsule polysaccharide biosynthesis
evm.model.sctg_0121_0002.16	CNVH-domain-containing protein
evm.model.sctg_0631_0001.1	Dipterans toxic crystal protein
evm.model.sctg_0013_0001.105	FAD NAD-binding domain-containing protein
evm.model.sctg_0013_0001.104	FAD NAD-binding domain-containing protein
evm.model.sctg_1050_0001.1	Gag protein
evm.model.sctg_0048_0006.5	Hemopexin domain-containing protein
evm.model.sctg_0344_0002.10	P-loop containing nucleoside triphosphate hydrolase protein
evm.model.sctg_0066_0001.15	Probable pectin lyase precursor
evm.model.sctg_0665_0003.1	Putative RecQ helicase
evm.model.sctg_0517_0002.1	Retrovirus-related pol polyprotein from transposon tnt 1-94
evm.model.sctg_1099_0001.3	Reverse transcriptase
evm.model.sctg_0028_0009.5	RNA polymerase Rpb1 C-terminal repeat domain-containing protein
evm.model.sctg_1043_0001.1	Volvatoxin A2 precursor

**Table 4:** Moniliophthora hemibiotrophic intersect

**AB intersect moniliophthora**	
**Gene Id**	**Gene annotation**
evm.model.sctg_0025_0003.30	3-carboxymuconate cyclase
evm.model.sctg_0188_0002.6	Acetamidase formamidase
evm.model.sctg_0009_0002.120	Alpha beta hydrolase fold protein
evm.model.sctg_0055_0002.11	Amidohydrolase
evm.model.sctg_0260_0001.15	Amidohydrolase
evm.model.sctg_0053_0001.15	Antibiotic biosynthesis monooxygenase
evm.model.sctg_0072_0001.2	Antibiotic biosynthesis monooxygenase
evm.model.sctg_0210_0002.9^N^	Antibiotic biosynthesis monooxygenase^N^
evm.model.sctg_0108_0002.35	Antibiotic biosynthesis monooxygenase domain protein
evm.model.sctg_0150_0003.5	Arabinofuranosidase
evm.model.sctg_0211_0001.12	Aryl sulfotransferase
evm.model.sctg_0005_0005.16^N^	Biotrophy-associated secreted protein 2^N^
evm.model.sctg_0066_0001.46^N^	Biotrophy-associated secreted protein 2^N^
evm.model.sctg_0104_0004.11	Biotrophy-associated secreted protein 2
evm.model.sctg_0099_0003.12	C6 zinc finger domain-containing protein
evm.model.sctg_0024_0001.46	Carbohydrate esterase family 12 protein
evm.model.sctg_0025_0003.31	Carbohydrate esterase family 12 protein
evm.model.sctg_0086_0003.12	Carbohydrate esterase family 12 protein
evm.model.sctg_0066_0001.40	Carboxyphosphonoenolpyruvate phosphonomutase-like protein
evm.model.sctg_0063_0003.8	Cell wall glycosyl hydrolase
evm.model.sctg_0155_0002.9	Cell wall glycosyl hydrolase
evm.model.sctg_0155_0004.12	Cell wall glycosyl hydrolase
evm.model.sctg_0155_0004.13^B^	Cell wall glycosyl hydrolase^B^
evm.model.sctg_0245_0001.21	Cellulose-binding family ii
evm.model.sctg_0176_0001.14^N^	Cellulose-binding protein^N^
evm.model.sctg_0243_0004.9	Cellulose-binding protein
evm.model.sctg_0065_0001.23	Chitin binding
evm.model.sctg_0068_0003.6	Chitin binding
evm.model.sctg_0086_0003.11	Chitin binding
evm.model.sctg_0086_0003.19	Chitin binding
evm.model.sctg_0149_0001.3	Cysteine-rich secreted protein
evm.model.sctg_0011_0002.81	duf1446 domain containing protein
evm.model.sctg_0073_0003.23	duf1446 domain containing protein
evm.model.sctg_0091_0003.16	duf567 domain protein
evm.model.sctg_0008_0001.9	duf718 domain protein
evm.model.sctg_0119_0001.2	duf985 domain protein
evm.model.sctg_0121_0002.8	endo- -beta-xylanase precursor
evm.model.sctg_0132_0002.1	Epoxidase subunit a
evm.model.sctg_0072_0002.6	Erylysin b
evm.model.sctg_0055_0003.16	Ethyl tert-butyl ether degradation
evm.model.sctg_0042_0004.3	Excitatory amino acid transporter 2
evm.model.sctg_0050_0004.25	Exonuclease
evm.model.sctg_0022_0002.37	Extracellular chitosanase
evm.model.sctg_0054_0001.12^N^	FAD binding domain protein^N^
evm.model.sctg_0133_0001.4	Feruloyl
evm.model.sctg_0015_0003.16	Fructose-bisphosphate aldolase
evm.model.sctg_0101_0001.1	Fusicoccadiene synthase
evm.model.sctg_0206_0002.8	General substrate transporter-like protein
evm.model.sctg_0214_0001.8	Glucokinase regulator family
evm.model.sctg_0011_0002.91^B^	Glycoside hydrolase family 16 protein^B^
evm.model.sctg_0011_0002.92	Glycoside hydrolase family 16 protein
evm.model.sctg_0091_0003.6	Glycoside hydrolase family 29 protein
evm.model.sctg_0120_0002.15	Glycoside hydrolase family 29 protein
evm.model.sctg_0195_0003.1	Glycoside hydrolase family 78 protein
evm.model.sctg_0063_0003.36	Glycosyl family
evm.model.sctg_0002_0008.34	Glycosyl hydrolase family 32
evm.model.sctg_0120_0001.8	Het domain containing protein
evm.model.sctg_0018_0003.8	Het domain protein
evm.model.sctg_0068_0003.29	Het domain protein
evm.model.sctg_0175_0002.5	Het domain protein
evm.model.sctg_0014_0004.20	Hydantoinase
evm.model.sctg_0008_0001.24	Hydroxycinnamoyl shikimate quinate hydroxycinnamoyltransferase
evm.model.sctg_0091_0003.1^N^	Integral membrane protein^N^
evm.model.sctg_0092_0002.20	Integral membrane protein
evm.model.sctg_0092_0002.22	Integral membrane protein
evm.model.sctg_0097_0001.11	Integral membrane protein
evm.model.sctg_0002_0010.51	Isochorismatase hydrolase
evm.model.sctg_0231_0001.20	Isoflavone reductase family protein
evm.model.sctg_0015_0001.8	Killer kp4 toxin
evm.model.sctg_0088_0001.8^N^	Lactam utilization protein^N^
evm.model.sctg_0023_0001.50	Lea domain protein
evm.model.sctg_0178_0001.5^N^	Lea domain-containing protein^N^
evm.model.sctg_0005_0005.46	Lipoprotein
evm.model.sctg_0049_0006.14	l-lysine −2,3-aminomutase
evm.model.sctg_0002_0010.48	Major facilitator superfamily transporter
evm.model.sctg_0240_0001.1	Major royal jelly protein
evm.model.sctg_0023_0002.33	mannitol-1-phosphate 5-dehydrogenase
evm.model.sctg_0158_0002.2	Metal dependent phosphohydrolase
evm.model.sctg_0081_0002.17^B^	NAD-binding phosphogluconate dehydrogenase-like protein^B^
evm.model.sctg_0110_0001.5	NAD-binding phosphogluconate dehydrogenase-like protein
evm.model.sctg_0062_0002.32	nmra-like protein
evm.model.sctg_0026_0001.19	Pectate lyase
evm.model.sctg_0214_0001.16	Pectate lyase
evm.model.sctg_0049_0006.8	Phosphoglycerate mutase family
evm.model.sctg_0049_0006.9	Phosphoglycerate mutase family
evm.model.sctg_0194_0001.15	Plasmid p 4b orf-3 family protein
evm.model.sctg_0094_0005.7	P-loop containing nucleoside triphosphate hydrolase protein
evm.model.sctg_0014_0004.25	Poxa3b laccase small subunit
evm.model.sctg_0162_0004.8	Proline racemase
evm.model.sctg_0018_0002.9	Proline-specific peptidase
evm.model.sctg_0089_0002.36	Protein tprxl
evm.model.sctg_0122_0004.1	Proteophosphoglycan ppg4
evm.model.sctg_0159_0004.4	Purine nucleoside
evm.model.sctg_0022_0002.64	Putative zinc metallopeptidase protein
evm.model.sctg_0100_0003.5	Related to tol protein
evm.model.sctg_0020_0001.61	Serine threonine sps1
evm.model.sctg_0217_0001.20	Surface cell-adhesion protein
evm.model.sctg_0105_0006.2^N^	Thaumatin-like protein^N^
evm.model.sctg_0100_0003.7	Tol-like protein
evm.model.sctg_0098_0001.12	Transcription regulator
evm.model.sctg_0093_0001.28^N^	Transferase family protein^N^
evm.model.sctg_0016_0005.17	Transposon en spm sub-class
evm.model.sctg_0188_0001.3	Twin-arginine translocation pathway signal
evm.model.sctg_0041_0004.5^N^	Urea hydro-lyase cyanamide^N^
evm.model.sctg_0079_0008.3	Variable surface lipoprotein d1
evm.model.sctg_0122_0001.3	ww rsp5 wwp

N indicates expression during the necrotrophic phase and B indicates expression during the biotrophic phase

**Table 5:** Pathogenic intersect

**ABE intersect pathogenic fungi**	
**Gene Id**	**Gene annotation**
evm.model.sctg_0099_0004.5	3-dehydroquinate dehydratase
evm.model.sctg_0005_0001.15	4-carboxymuconolactone decarboxylase
evm.model.sctg_0024_0002.8	4-hydroxyphenylpyruvate dioxygenase
evm.model.sctg_0220_0007.7	Acetyltransferase
evm.model.sctg_0108_0002.26	Arylsulfatase
evm.model.sctg_0002_0010.39	Cupin domain protein
evm.model.sctg_0004_0002.40	Cytochrome c oxidase assembly protein
evm.model.sctg_0029_0001.27	Extracellular invertase
evm.model.sctg_0040_0001.46	Flavin-containing amine oxidasedehydrogenase
evm.model.sctg_0006_0005.98	Formate nitrite transporter
evm.model.sctg_0084_0003.26	GAF domain nucleotide-binding protein
evm.model.sctg_0289_0002.8	Glycoside hydrolase family 45 protein
evm.model.sctg_0089_0002.33	gnat family acetyltransferase
evm.model.sctg_0185_0001.1	gpi-anchored protein
evm.model.sctg_0040_0003.2	Haloacid dehalogenase type ii
evm.model.sctg_0346_0003.4	Tubulin
evm.model.sctg_0011_0002.41	Iron permease ftr1
evm.model.sctg_0309_0002.8	Minor histocompatibility antigen h13
evm.model.sctg_0002_0009.45	Nitrilase 4
evm.model.sctg_0018_0008.47	Nucleoside-diphosphate-sugar
evm.model.sctg_0018_0007.18	Phytanoyl-CoA dioxygenase family protein
evm.model.sctg_0042_0011.14	Related to transposase
evm.model.sctg_0046_0002.12	rpel repeat protein
evm.model.sctg_0073_0003.11	Secreted hydrolase
evm.model.sctg_0354_0001.6	sirq protein
evm.model.sctg_0020_0001.23	spherulin 4-like cell surface protein
evm.model.sctg_0162_0003.2	Taurine catabolism dioxygenase
evm.model.sctg_0105_0006.1	Thaumatin-like protein 1-like
evm.model.sctg_0006_0005.105	Transcriptional regulator
evm.model.sctg_0138_0001.17	upf0132 domain protein

**Table 6:** Biotrophic intersect

**ABC intersect biotrophic fungi**	
**Gene Id**	**Gene annotation**
evm.model.sctg_0063_0001.9	alpha- -mannosylglycoprotein 6-beta-n-acetylglucosaminyltransferase a
evm.model.sctg_0124_0001.10	Aminoglycoside phosphotransferase
evm.model.sctg_0007_0001.162	Dolichol phosphate-mannose biosynthesis regulatory protein
evm.model.sctg_0112_0002.7	dolichyl-phosphate mannosyltransferase polypeptide 3
evm.model.sctg_0040_0002.2	gas1-like protein
evm.model.sctg_0016_0002.4	Glycoside hydrolase family 61 protein
evm.model.sctg_0063_0002.2	Glycoside hydrolase family 95 protein
evm.model.sctg_0001_0002.138	Hydantoinase
evm.model.sctg_0919_0001.1	Hypothetical proline-rich protein
evm.model.sctg_0166_0004.8	Nuclear fusion protein kar5
evm.model.sctg_0041_0003.8	Proteophosphoglycan 5
evm.model.sctg_0231_0001.2	Response regulator receiver domain-containing protein
evm.model.sctg_0229_0001.10	Ribosomal protein l36 containing protein
evm.model.sctg_0243_0004.11	Transcription factor iib
evm.model.sctg_0005_0002.50	Transcriptional family alpha beta fold family protein
evm.model.sctg_0096_0003.37	Transmembrane protein 167a
evm.model.sctg_0003_0004.13	Vacuolar h + −atpase assembly protein

### Identification of secreted proteins

Because of the hemibiotrophic nature of *M. roreri* and the role that secreted proteins are expected to play in that interaction, we analyzed the genome for genes that express secreted proteins. The first screening identified all of the putative proteins with signal peptides. In *M. roreri* 1,752 proteins (9.7%) were found to have signal peptides, which is slightly lower than *M. perniciosa* which has 1,810 proteins with signal peptides. Next the proteins with signal peptides were analyzed for transmembrane domains, to remove proteins that are expected to be incorporated into the membrane of the fungus. Ninety-two of the 1,752 proteins have two or more transmembrane domains. One hundred and twenty-five glycosylphosphatidylinositol (GPI) anchor proteins were also excluded from the list of secreted proteins (Table 7). While the GPI-anchored proteins are normally removed from secreted proteins, the posttranslational modification of glypiation is the only method of attachment to the membrane for these proteins, and because the bonds are subject to phospholipase cleavage, these proteins could still be released into the extracellular space via an enzymatic release process. After removal of proteins with transmembrane and GPI moieties the total number of *M. roreri* secreted proteins was 1,535, which is less than the 1,596 secreted proteins predicted for *M. perniciosa*.

**Table 7:** Results of the genome based secretome analysis

	**total # of predicted proteins**	**Protein without signal peptides**	**Proteins with signal peptides**	**Proteins with more than 2 transmembrane domains**	**Proteins with GPI domains**	**Total number of secreted proteins**	**% proteins with signal peptides**	**Unique signal peptides**
						**Minus TMD and GPI domains**		
*M. roreri*	17920	16167	1752	92	125	1535	9.7	39
*M. perniciosa*	17016	15206	1810	92	122	1596	10.6	58

### Expression of the secretome

In this study we wanted to verify the expression of the predicted genes, including the secretome identified in the genome analysis. We utilized a replicate set of infected pod tissues from a previous study to obtain transcriptomes representing the biotrophic and necrotrophic phases of the disease [21]. While our present focus is on the secreted proteins, the complete fungal transcriptome, for FPR, in different resistant plant backgrounds will be addressed in a separate treatise. The planting material used represents a segregating F1 population of progeny from highly susceptible clones (PA16 × SIC433 and PA16 × EEG29). Briefly, the pods used in this study were derived from flowers hand-pollinated 60 days prior to infection with a spore suspension of 1×10^8^*M. roreri* spores ml^-1^. The infected pods were frozen in liquid nitrogen and lyophilized at 30 days post infection (DPI) and at 60 DPI. Based on typical progression of FPR symptoms [21], three malformed green pods were selected for RNA-Seq and RT-qPCR from 30 DPI pods (biotrophic phase) and three necrotic sporulating pods were selected for RNA-Seq and RT-qPCR from 60 DPI pods (necrotrophic phase).

The 30 DPI pod samples yielded 67,818,927; 64,099,928 and 64, 033,574 reads and the number of fungal reads per sample were 149,675 (0.22%), 122,289 (0.19%) and 312,684 (0.49%), respectively. For the 60 DPI pod samples 73,037,363; 66,395,887 and 69,434,845 reads were sequenced, with fungal reads accounting for 603,498 (0.83%), 882,821 (1.33%) and 503,459 (0.73%), respectively. When all of the libraries were compared 13,759 genes (77% of the predicted CDS) from the *M. roreri* genome were found to be expressed in these infected pods. The secreted proteins were then separated from the other expressed genes. Among the 1,535 secreted protein genes identified 1,355 or 88%, were expressed at some level under the tested conditions. Using a P-value of 0.05, we found that 222 of the secreted protein genes were differentially expressed between the 30 DPI and 60 DPI pods. All of the expressed secreted protein genes are listed in Additional file 2. The data revealed phase-dependent differential gene expression, where 164 of the genes were up-regulated in the 60 DPI pods while 58 were upregulated in the 30 DPI pods. Since the hypothetical genes can provided no information regarding their putative function they were eliminated from further consideration with regards to their role in the disease process.

Genes expressed during the early stages of infection and disease development reflect gene products that function in the biotrophic phase of the disease development, which can be initially asymptomatic. Late-stage gene expression yields gene products that function in the necrotrophic phase, which is associated with pod chlorosis and necrosis, and possibly with sporulation of the fungus on pod tissue. To validate the differential expression of genes encoding secreted proteins as suggested by the RNA-Seq analysis, RT-qPCR was conducted, with a selected number of genes comparing expression in a set of seven malformed green pods at 30 DPI and seven necrotic pods at 60 DPI. At 30 DPI, 34 genes encoding secreted proteins with putative functions were expressed, with one being expressed only at this stage (Table 8). At 60 DPI, 105 genes encoding predicted secreted proteins with putative functions were expressed (Table 9). Forty-one of the 105 60 DPI genes were detected only in the necrotrophic phase. RT-qPCR confirmed the up-regulation of the 30 DPI and 60 DPI genes (Tables 8 and 9). Although the GPI-anchored proteins were removed from the secretome we did find a number of them highly expressed in both the biotrophic and necrotrophic phases. Table 10 provides a list of the most highly expressed genes encoding GPI-anchored proteins in both phases since controlled release of these proteins from the fungal membrane is possible.

**Table 8:** Biotrophic gene Expression of putative Secreted Proteins

**Transcript Id**	**Gene annotation**	**RNAseq**					**QPCR**			
		**30DPI**	**60DPI**	**Pvalue**	**Fold change**	**Primers**	**Induced 30-60 DPI**		**Repressed 30-60 DPI**	
		Mean*	Mean*				**Mean**	**St. E.**	**Mean**	**St. E.**
e.m.s_0369_0002.2	Acetylxylan esterase	638.4	91.6	5.8E-03	7.0					
e.m.s_0461_0001.1	Carbohydrate esterase family 8 protein	512.1	67.0	8.8E-04	7.6	MrCE8a	20.868	8.9736		
e.m.s_0215_0001.21	Carbonic anhydrase	16.3	0.4	2.2E-02						
e.m.s_0250_0003.8	Catalase cat1	294.9	59.3	6.4E-03	5.0	MrCAT1	114.906	48.407		
e.m.s_0155_0004.13	Cell wall glycosyl hydrolase	330.4	26.3	7.4E-05	12.6					
e.m.s_0192_0001.1	Cerato-platanin	3014.0	520.7	5.3E-03	5.8					
e.m.s_0192_0003.14	Cerato-platanin	4931.3	968.5	7.2E-03	5.1					
e.m.s_0192_0003.17	Cerato-platanin	7602.6	912.5	7.1E-04	8.3					
e.m.s_0258_0001.1	Defense-related protein scp domain-containing protein MrPR-1 g	16778.8	1200.4	5.6E-04	14.0	MrSCP	434.018	253.661		
e.m.s_0251_0004.1	Defense-related protein scp domain-containing protein MrPR-1i2	32.6	2.4	7.4E-02						
e.m.s_0516_0001.1	Defense-related protein scp domain-containing protein Mr-PR-1n	499.0	13.7	2.6E-06	38.3					
e.m.s_0041_0005.1	Endo-polygalacturonase	2621.4	32.4	1.4E-06	81.9	MrendoPG	911.453	592.910		
e.m.s_0407_0004.2	Galactan 1,3-beta-galactosidase	37.6	52.2	2.4E-02						
e.m.s_0027_0002.19	Galactose 1-epimerase	154.2	47.4	3.9E-02	3.3					
e.m.s_0011_0002.91	Glycoside hydrolase family 16 protein	1276.8	232.0	5.0E-03	5.5					
e.m.s_0030_0003.33	Glycoside hydrolase family 18 protein	6125.5	948.8	3.4E-03	6.5	MrGH18g	38.900	16.126		
e.m.s_0044_0001.33	Glycoside hydrolase family 18 protein	527.3	56.3	7.7E-04	9.4	MrGH18d	253.832	183.817		
e.m.s_0044_0001.6	Glycoside hydrolase family 18 protein	12287.0	3336.6	2.1E-02	3.7					
e.m.s_0114_0002.6	Glycoside hydrolase family 18 protein	12684.6	687.6	5.1E-06	18.5	MrGH18c	151.387	51.810		
e.m.s_0416_0001.5	Glycoside hydrolase family 18 protein	4897.1	266.4	3.1E-06	18.4					
e.m.s_0055_0003.9	Glycoside hydrolase family 28 protein	129.2	20.9	1.3E-02	6.1					
e.m.s_0067_0006.17	Glycoside hydrolase family 43	234.2	54.4	2.2E-02	4.3					
e.m.s_0043_0001.2	Glycoside hydrolase family 5 protein	3971.1	188.7	1.2E-06	21.1					
e.m.s_0531_0001.4	Glycoside hydrolase family 5 protein	3387.9	716.4	1.2E-02	4.7	MrGH5	69.7456	41.581		
e.m.s_0351_0003.10	Glycoside hydrolase family 92 protein	2935.9	894.7	4.6E-02	3.3					
e.m.s_0346_0003.2	Hydrophobin 3	431.5	65.6	1.8E-03	6.6	MrSc3P3	345.0789	196.349		
e.m.s_0346_0003.1	Hydrophobin sc3-like	6461.8	471.4	3.8E-05	13.7	MrSc3P2	356.391	177.576		
e.m.s_0409_0005.4	Hypothetical fad dependent oxidoreductase	566.2	119.8	1.4E-02	4.7					
e.m.s_0561_0002.1	Immunomodulatory protein	7180.5	746.5	3.2E-04	9.6					
e.m.s_0095_0005.5	Major facilitator superfamily multidrug-resistance	7.0	0.0	1.3E-02						
e.m.s_0053_0004.6	Membrane autotransporter barrel domain protein	755.7	203.5	2.5E-02	3.7	MrBDP	5.184	1.528		
e.m.s_0094_0001.8	O-methyltransferase	1131.7	488.6	8.2E-04						
e.m.s_0064_0001.5	Tryptophan dimethylallyltransferase	190.8	0.9	3.2E-04	211.1	MrTRY-DMA	235.864	134.155		
e.m.s_0039_0002.43	WSC domain protein	356.0	102.1	4.1E-02	3.5					
e.m.s_0478_0002.2	Xylanase A	1135.8	9.0	9.3E-04	126.0	MrXYLa	171.086	77.159		

Mean* is the normalized mean based on the size factor of the libraries.

**Table 9:** Necrotrophic genes expression of putative Secreted proteins

**Transcript Id**	**Gene annotation**	**RNAseq**					**QPCR**			
		**30DPI**	**60DPI**	**Pvalue**	**Fold change**	**Primers**	**Induced 30-60 DPI**		**Repressed 30-60 DPI**	
		Mean*	Mean*				**Mean**	**St. E.**	**Mean**	**St. E.**
e.m.s_0228_0004.4	Alpha beta hydrolase	3.2	34.5	1.4E-02	10.8					
e.m.s_0245_0001.13	Alpha1,3-glucanase/mutanase	0.7	171.8	8.3E-03	245.4					
e.m.s_0185_0001.10	Aryl-alcohol oxidase	0.0	21.4	5.7E-04	21.4					
e.m.s_0185_0001.9	Aryl-alcohol oxidase	0.0	7.8	4.2E-02	7.8					
e.m.s_0274_0001.9	Aryl-alcohol oxidase	0.0	9.6	2.1E-02	9.6					
e.m.s_0491_0001.5	Aryl-alcohol oxidase	3.9	61.7	3.0E-02	15.9					
e.m.s_0100_0003.8	Arylsulfatase	2.6	42.4	2.9E-02	16.4					
e.m.s_0251_0003.2	Aspartic peptidase a1	11.5	46.7	3.6E-02	4					
e.m.s_0056_0003.12	Aspartic-type endopeptidase	0.7	25.6	2.5E-03	36.6					
e.m.s_0463_0001.8	Beta-lactamase class penicillin binding protein	6.6	324.6	1.5E-03	492.4					
e.m.s_0066_0001.46	Biotrophy-associated secreted protein 2 MrBAS1	7.6	496.7	1.0E-03	66.1	MrSP2a			26.509	15.802
e.m.s_0005_0005.16	Biotrophy-associated secreted protein 2 MrBAS2	2.0	613.6	3.2E-04	322.6					
e.m.s_0227_0001.20	Calcineurin-like phosphoesterase	1.8	19.0	9.5E-03	10.6					
e.m.s_0066_0001.21	Carbohydrate esterase family 4 protein	0.7	83.8	1.8E-02	119.7					
e.m.s_0155_0002.4	Carbohydrate esterase family 4 protein	0.0	50.5	2.1E-06	50.5					
e.m.s_0008_0001.25	Carbonic anhydrase	0.7	121.3	7.0E-08	172.8					
e.m.s_0190_0004.1	Carboxypeptidase cpds	3.8	460.0	4.4E-07	121.1					
e.m.s_0190_0004.2	Carboxypeptidase cpds	3.1	28.5	5.1E-03	9.2					
e.m.s_0176_0001.14	Cellulose-binding protein	0.0	22.2	4.7E-04	22.2					
e.m.s_0058_0002.19	Cerato platanin	0.0	16.2	3.2E-03	16.2					
e.m.s_0058_0002.21	Cerato-platanin	2.1	36.2	2.7E-02	18.1					
e.m.s_0192_0001.2	Cerato-platanin	0.0	20.7	7.5E-04	20.7					
e.m.s_0192_0001.6	Cerato-platanin	0.0	494.9	2.2E-04	494.9					
e.m.s_0297_0002.3	Chitin deacetylase family 4	0.0	40.4	2.8E-02	40.5	MrCHIDACTb			5663.095	3460.583
e.m.s_0245_0001.24	Chitin synthase	0.0	2158.9	2.8E-04	2159	MrCHS			2515.177	1862.483
e.m.s_0008_0004.19	Copper amine oxidase	4.3	77.0	6.6E-03	17.9					
e.m.s_0003_0001.85	Cutinase	0.0	9.5	2.3E-02	9.5					
e.m.s_0017_0003.61	Cytochrome p450	0.0	32.2	9.1E-03	32.2					
e.m.s_0052_0004.10	Cytochrome p450	0.7	14.9	3.2E-02	21.1					
e.m.s_0470_0001.6	Cytochrome p450 monooxygenase	5.0	91.2	1.1E-04	18.3	MrCp450a			30.050	11.8905
e.m.s_0059_0001.14	Deuterolysin m35 metalloprotease	2.8	137.0	5.0E-02	48.9					
e.m.s_0099_0003.13	Dioxygenase family protein	8.3	5904.3	3.6E-02	715.6					
e.m.s_0061_0002.41	Exo-beta 1,3-glucanase	2.6	16.7	4.4E-02	6.4					
e.m.s_0026_0005.53	Expansin family protein	0.0	1165.0	9.8E-04	1165	MrEXP-A			326.810	159.020
e.m.s_0086_0003.2	Expansin family protein	1.8	37.3	2.6E-04	20.7					
e.m.s_0280_0002.5	Extracellular dioxygenase	0.0	15.1	3.4E-03	15.1					
e.m.s_0107_0002.16	Extracellular triacylglycerol lipase precursor	0.0	9.8	2.0E-02	9.8					
e.m.s_0107_0002.18	Extracellular triacylglycerol lipase precursor	0.0	9.8	2.3E-02	9.8					
e.m.s_0352_0003.3	Extracellular triacylglycerol lipase precursor	0.0	9.7	2.1E-02	9.7					
e.m.s_0054_0001.12	FAD binding domain protein	31.7	306.9	2.3E-04	9.7					
e.m.s_0135_0001.27	FAD binding domain-containing protein	0.0	43.9	6.4E-06	43.9					
e.m.s_0277_0002.12	FAD binding domain-containing protein	6.5	91.2	1.1E-04	14.1					
e.m.s_0277_0002.13	FAD binding domain-containing protein	2.6	124.8	1.1E-04	48					
e.m.s_0375_0001.9	FAD binding domain-containing protein	2.0	85.8	9.5E-06	43.1					
e.m.s_0459_0001.6	FAD binding domain-containing protein	0.0	36.5	3.2E-04	36.5					
e.m.s_0469_0001.4	FAD binding domain-containing protein	0.0	8.4	3.7E-02	8.4					
e.m.s_0002_0010.20	F-box and fnip repeat-containing protein	114.0	3336.6	2.1E-02	29.3					
e.m.s_0087_0002.1	Fruit-body specific gene a	0.7	20.9	7.7E-03	29.8					
e.m.s_0053_0004.14	Fungal peroxidase	3.6	22.3	1.5E-02	6.3					
e.m.s_0111_0006.5	Glucooligosaccharide oxidase	0.0	27.8	8.3E-03	27.8					
e.m.s_0211_0001.14	Glucose oxidase	0.0	66.0	3.6E-07	66					
e.m.s_0085_0002.37	Glycoside hydrolase family 16 protein	0.0	30.0	7.7E-05	29					
e.m.s_0021_0002.67	Glycoside hydrolase family 18 protein	19.6	87.3	5.0E-02	4.5					
e.m.s_0261_0004.2	Glycoside hydrolase family 3 protein	25.4	127.4	1.7E-02	5					
e.m.s_0008_0004.3	Glycoside hydrolase family 30 protein	11.7	67.7	3.7E-02	6					
e.m.s_0232_0001.2	Glycoside hydrolase family 35 protein	33.9	128.7	4.3E-02	3.8					
e.m.s_0438_0002.3	Glycoside hydrolase family 35 protein	0.0	19.7	8.7E-04	19.7					
e.m.s_0007_0001.127	Glycoside hydrolase family 5 protein	27.1	163.4	5.6E-03	6					
e.m.s_0265_0002.8	Glycoside hydrolase family 5 protein	10.8	111.9	5.4E-05	10.4					
e.m.s_0241_0001.11	Glycoside hydrolase family 61 protein	0.0	26.6	9.6E-03	26.6					
e.m.s_0241_0001.12	Glycoside hydrolase family 61 protein	0.0	43.1	5.9E-03	43.1					
e.m.s_0155_0002.8	Glycoside hydrolase family 61 protein	13.5	115.5	1.3E-02	8.6					
e.m.s_0004_0003.50	Glycoside hydrolase family 9 protein	10.4	63.2	8.9E-03	6.3	MrGH9			4.311	1.827
e.m.s_0200_0003.3	Glycosyl hydrolase family 10	0.7	22.6	3.6E-02	32.2					
e.m.s_0333_0001.1	Glyoxal oxidase	4.9	47.9	5.1E-03	9.8					
e.m.s_0135_0001.28	gmc oxidoreductase	14.3	52.2	2.4E-02	3.6					
e.m.s_0074_0003.10	Hemerythrin hhe cation binding domain-containing protein	49.9	383.1	2.1E-02	7.7					
e.m.s_0039_0002.15	Hemolysin	0.7	16.1	2.3E-02	22.9					
e.m.s_0062_0001.18	Heterokaryon incompatibility protein het-c	28.2	116.3	1.5E-02	4.1					
e.m.s_0018_0008.40	Hydrophobin	13.2	86.6	3.6E-02	6.6					
e.m.s_0018_0008.54	Hydrophobin	0.7	61.9	1.2E-05	87.1					
e.m.s_0018_0008.56	Hydrophobin	0.0	256.2	4.3E-12	256	MrLM18			1573.702	1268.272
e.m.s_0058_0002.53	Hydrophobin	0.7	25.1	2.8E-03	35.7					
e.m.s_0058_0002.55	Hydrophobin	9.0	304.7	5.8E-07	34.2					
e.m.s_0149_0001.14	Hydrophobin	0.0	11.2	1.2E-02	11.2					
e.m.s_0149_0001.16	Hydrophobin	0.0	425.5	2.3E-06	425.6	MrLM19			257.737	133.646
e.m.s_0170_0002.12	Hydrophobin	0.0	9.3	2.4E-02	9.3					
e.m.s_0298_0001.2	Hydrophobin	0.0	11.4	1.2E-02	11.4					
e.m.s_0149_0001.15	Hydrophobin 2	0.0	205.6	1.6E-04	205.6					
e.m.s_0199_0001.3	Laccase	0.0	9.6	2.6E-02	9.6					
e.m.s_0210_0002.8	Laccase	0.0	119.6	1.0E-06	119.6					
e.m.s_0246_0002.7	Laccase	1.3	41.0	1.0E-02	31.8					
e.m.s_0279_0002.3	Laccase	0.0	21.1	3.4E-02	21.2					
e.m.s_0082_0001.16	Mannoprotein	88.8	298.1	4.7E-02	3.4					
e.m.s_0030_0003.64	Metalloproteinase	18.6	306.9	8.1E-06	16.5					
e.m.s_0406_0002.4	Necrosis inducing-like protein npp1 type	9.1	658.2	1.5E-04	73.1	MrNPP1			310.949	181.890
e.m.s_0051_0003.3	nhl repeat-containing protein	0.0	28.8	1.1E-04	28.8					
e.m.s_0120_0002.7	Oxalate decarboxylase	9.3	57.9	4.8E-02	6.2					
e.m.s_0398_0002.11	Oxidoreductase fad binding	1.8	111.0	5.4E-04	61.6					
e.m.s_0639_0001.6	Para-nitrobenzyl esterase	0.0	45.8	3.2E-04	45.8					
e.m.s_0082_0001.36	Peptidase m28	3.2	57.6	3.9E-02	18					
e.m.s_0007_0001.138	Peptide-n4-(n-acetyl-beta-glucosaminyl)asparagine amidase a	0.0	12.6	3.5E-02	12.5					
e.m.s_0180_0002.16	Phosphatidylserine decarboxylase	4.3	451.8	5.1E-04	105.1					
e.m.s_0432_0001.5	PR-1 protein MrPR-1d	5.8	45.1	1.4E-03	7.8					
e.m.s_0568_0001.7	Proline-rich antigen	7.7	1372.5	7.7E-03	177.3					
e.m.s_0018_0003.1	Proline-specific peptidase	29.2	94.0	5.0E-02	3.2					
e.m.s_0726_0001.2	Pyrolysin	8.1	50.9	3.5E-02	6.3					
e.m.s_0125_0001.43	Riboflavin aldehyde-forming enzyme	61.2	229.9	3.8E-02	3.8					
e.m.s_0040_0001.30	Ribonuclease t1	0.0	11.4	1.1E-02	11.4					
e.m.s_0321_0001.8	Serine-rich protein	16.4	289.5	5.2E-04	18.1	MrSRP			14.072	5.613
e.m.s_0005_0005.47	Serine-type endopeptidase	224.0	811.1	4.1E-02	3.6	MrS-endo			3.482	0.866
e.m.s_0021_0002.36	Tripeptidyl peptidase A	0.0	20.8	6.5E-04	20.8					
e.m.s_0166_0004.10	Tripeptidyl peptidase A	1.8	39.5	1.8E-04	21.9					
e.m.s_0048_0003.5	Virus P4 KP4 toxin	0.0	118.9	4.5E-02	119	MrKP4			134.442	52.042
e.m.s_0014_0002.14	wsc domain-containing protein	0.0	42.6	8.7E-06	42.6					
e.m.s_0010_0002.57	Zinc metalloprotease	10.7	92.8	2.6E-03	8.7					

Mean* is the normalized mean based on the size factor of the libraries.

**Table 10:** GPI anchor proteins

**Gene Id**	**Gene name**	**30DPI**	**60 DPI**	**Pvalue**
		Mean*	Mean*	
evm.model.sctg_0115_0006.6	Related to tgf beta induced protein ig-h3 precursor	1303.93	283.09	1.03E-02
evm.model.sctg_0044_0002.3	Chitin deacetylase 9	330.81	98.49	4.34E-02
evm.model.sctg_0035_0002.62	gmc oxidoreductase	15.03	10883.07	3.48E-03
evm.model.sctg_0474_0002.6	gmc oxidoreductase	1.99	6162.70	1.85E-04
evm.model.sctg_0154_0003.4	Nucleus protein	21.52	377.01	1.01E-05
evm.model.sctg_0023_0002.46	Aspartic peptidase a1	5.89	793.02	5.90E-05
evm.model.sctg_0233_0003.16	gmc oxidoreductase	12.78	268.23	9.28E-03
evm.model.sctg_0090_0005.3	Aspartic peptidase a1	4.60	105.14	4.91E-05
evm.model.sctg_0003_0001.29	Carbohydrate esterase family 4 protein	81.65	240.08	4.51E-02
evm.model.sctg_0470_0001.4	Carbohydrate esterase family 4 protein	23.70	94.23	1.66E-02
evm.model.sctg_0007_0001.101	Extracellular serine-rich	264.05	1547.88	2.37E-03
evm.model.sctg_0016_0004.31	Glycoside hydrolase family 16 protein	0.70	17.08	1.66E-02
evm.model.sctg_0061_0002.53	Glycosyl hydrolase 53 domain-containing protein	2.80	87.42	2.18E-02
evm.model.sctg_0069_0001.19	Glyoxal oxidase	15.69	92.15	3.58E-02
evm.model.sctg_0233_0003.16	gmc oxidoreductase	12.78	268.23	9.28E-03
evm.model.sctg_0149_0001.6	Macrofage activating glycoprotein	5.38	47.41	4.89E-02
evm.model.sctg_0149_0001.5	Macrofage activating glycoprotein	1.99	59.62	1.07E-04
evm.model.sctg_0154_0003.4	Nucleus protein	21.52	377.01	1.01E-05
evm.model.sctg_0020_0001.30	Serine-threonine rich	92.38	614.18	4.02E-03

Mean* is the normalized mean based on the size factor of the libraries.

Several gene families of secreted proteins were prominently upregulated in the biotrophic phase. In this phase the 34 genes with known functions were primarily associated with nutrient acquisition and with plant and fungal cell wall modification. The largest single group identified was glycoside hydrolases (GH). Eleven glycoside hydrolases genes were upregulated: two genes from GH family 5; five genes from GH family 18; and one gene each from the GH families 16, 28, 43, 92. The GH family 18 genes are homologous to chitinase genes (EC 3.2.1.14) [22] and GH family 5 genes are endo-enzymes that are capable of hydrolyzing both β-mannans and β-glucans [23] and are homologous to beta-1,3-glucanase, which includes endoglucanase, beta-mannanase, exo-1,3-glucanase, endo-1,6-glucanase, xylanase, and endoglycoceramidase. The GH 16 s are homologous to endobeta 1,3-glucanases (EC 3.2.1.39) and xyloglucan:xylogluosyltransferase (EC 2.4.1.207) [24], GH 28 s are homologous to polygalacturonases and includes pectin degrading enzymes like polygalacturonase (EC 3.2.1.15), exopolygalacturonase (EPG; EC 3.2.1.67), exo-poly-α-galacturonosidase (EC3.2.1.82) [25], GH 43 s are homologous to beta-xylosidases (EC 3.2.1.37) [26], while the GH 92 s are alpha-1,2–mannosidases [27] (Additional file 2). In addition to the GHs, other enzymes were identified such as galactose-1-epimerase, a carbonic anhydrase, a family 8 carbohydrate esterase (pectin methylesterase), and a tryptophan dimethylally transferase. The plant cell wall-modifying enzymes identified in the biotrophic phase include an endo-polygalacturonase, a galactan 1,3-beta-galactosidase, a cell wall glycosyl hydrolase, a catalase, an acetylxylan esterase and a xylanase A. Additionally, three cerato-platanin genes, two hydrophobins, three PR-1-like proteins, and an immunomodulatory protein were all induced in the biotrophic phase that could be involved in the fungal host interaction (Table 8).

Based on RNA-Seq and verified by RT-qPCR analysis, the majority of the genes within the 105 genes expressed during the necrotrophic phase are associated with fungal growth/pathogenicity and nutrient acquisition. Several genes families were identified with multiple expressed genes such as hydrophobins (10 genes), FAD binding domain containing proteins (6 genes), aryl-alcohol oxidases (4 genes), laccases (4 genes), extracellular triacylglycerol lipases (3 genes) and cerato-platanins (3 genes). Several of these highly upregulated genes are associated with lignin breakdown. The aryl-alcohol oxidases (AAOs) are FAD containing extracellular enzymes in the glucose-methanol choline oxidase (GMC) family [28]. AAOs are involved in lignin degradation where the enzyme interacts with p-coumaryl alcohol, coniferyl alcohol and sinapyl alcohol polymers that make up the lignin, and O_2_ yielding aromatic aldehydes and hydrogen peroxide [28]. Laccases are polyphenol oxidases that reduce phenolic compounds in lignin [29]. Glyoxal oxidases are extracellular enzymes that form hydrogen peroxide during the breakdown of lignin [30]. Eight GH families were identified among the 12 GH genes (two GH family 35; two GH family 61; two GH family 5 and one gene each for families 3, 9, 10, 16 and 18). The GH 35 s proteins are homologous to beta-galactosidases and GH 61 s proteins are homologous to endoglucanases. The GH 3 s proteins are homologous to beta–glucosidases; GH 9 s proteins are endoglucanases or cellulases; and GH 10s proteins are xylanases [31]. Numerous other enzymes and pathogenicity-related genes were identified such as carboxypeptidases, carbohydrate esterases, tripeptidyl peptidase, a necrosis inducing protein, a fungal peroxidase, cytochrome p450s, and expansins. The highest relative expressions were found for a dioxygenase, a Fbox and FNIP repeat-containing protein, a chitin synthase, a proline-rich antigen and one of the expansins (Table 9).

Several gene families were found to be differently regulated and had specific members upregulated in the biotrophic phase and others upregulated in the necrotrophic phase. Of the 41 hydrophobin genes found in the genome, 12 were differentially expressed; 10 were upregulated in the necrotrophic phase; two in the biotrophic phase. Among the eight cerato-platanin genes found in the genome six were differentially upregulated; three in each phase. The expression of genes encoding fungal-derived pathogenesis-related proteins was detected in samples from the biotrophic phase, with *MrPR-1n*, *MrPR-1 g* and *MrPR-1i2* being upregulated. Among the other nine genes encoding PR-1-like proteins found in the *M. roreri* genome, one *MrPR-1d* was upregulated in the necrotrophic phase and five were constitutively expressed under the tested conditions (Additional file 2).

## Discussion

### Genome comparison

We have produced a high-quality draft genome of *M. roreri* using different next-generation sequencing technologies. After sequence assembly using the whole genome shotgun (WGS) strategy, a total of 3,298 contigs with a N50 value of 48.1 kb was assembled with a total genome size calculated to be 52.3 Mbp. Although larger, the *M. roreri* genome is structurally and organizationally similar to *M. perniciosa,* a sister taxa with a draft genome consisting of 3,087 contigs (N50 = 48 kb) and a genome size of 44.7 Mbp. The majority of the size difference between the genomes is associated with repetitive DNA. The *M. roreri* genome size is larger than the genome of *M. perniciosa*, but the number of genes located in each genome is similar.

One factor for the size difference between *M. roreri* and *M. perniciosa* is highly correlated to the difference in the number of Long Terminal Repeat–Transposable Elements (LTR-TEs) between the genomes. About 3.46 Mbp or approximately 7% of the *M. roreri* genome is attributed to LTR-TEs located in the genome, while only 0.332 Mbp or about 1% of the *M. perniciosa* genome is attributed to LTR-TEs. Transposable Elements (TEs) contribute no more than 10 to 15% in most fungal taxa [32]. For example, the *Saccharomyces cereviseae* genome contains 3.1% TEs [33], the *Magnaporthe oryzae* genome ranges from 8.2-14% [34], while 24% of the *Laccaria bicolor* genome is transposon derived [35]. Within the *M. perniciosa* genome these repetitive elements are active and appear to contribute to the genetic variability of the species [20]. Another factor contributing to the size difference between the genomes is associated with repetitive elements and low complexity DNA. *M. roreri* genome has four times as many repetitive elements (7,060,129 bp) as found in the *M. perniciosa* genome (1,737,865 bp)*.* The classification and biological roles of these repetitive sequences and transposable elements in *M. roreri* are beyond the scope of this discussion but will be explored in depth in a separate treatise.

The high degree of similarity between *M. roreri* and *M. perniciosa* genomes supports their taxonomic relatedness, while distinctions have been identified that could contribute to their distinctive functional and morphologic differences. Bidirectional blast analysis of *M. roreri* with four other basidiomycetes revealed a set of 1,133 genes specific to *M. roreri*. The majority of these genes are hypothetical with only 16 genes having known homologies and most of these relate to TEs. Expression analysis of the A gene set (genes specific to *M. roreri*) revealed that only seven of these genes are expressed and all at low levels. Among the genes encoding hypothetical proteins that are specific to *M. roreri,* 129 were constitutively expressed and only three were differentially expressed in both the biotrophic and necrotrophic phases (Additional file 1, A set sheet). Low to mid-level constitutive expression in genes from the ABE and ABC intersects (Tables 5 and 6) suggest that these genes are used in general functions common to the sampled basidiomycetes such as metabolism. The differential expression of the 14 genes shared by *M. roreri* and *M. perniciosa* (AB intersect) in the biotrophic and necrotrophic phases, as expression of these genes suggests similarities in the hemibiotrophic life cycle of these species.

### Secretome targeting the plant cell

RNA-Seq, was used to validate the genome CDS through transcriptome expression analysis of *M. roreri* infected cacao pods at 30 DPI and 60 DPI. These two sampling points compare genes expressed in the early stage or biotrophic phase and the late stage or necrotrophic phase of the FPR disease cycle. *In silico* analysis of genes with signal peptides, which have a high likelihood of being transported outside of the fungus, yielded 1,355 genes expressed at some level in the 30 and 60 DPI samples, as detected by RNA-Seq. Only 222 of these genes were differentially expressed between the biotrophic and necrotrophic phases, using a P-value of 0.05. The similarities in gene expression between the two phases are greater than the dissimilarities. For example, genes encoding plant cell wall-degrading cellulases were not upregulated in the biotrophic phase, but, their expression was detected. Although the functions of many of these genes are unknown their expression raises the potential that they serve critical roles in the *M. roreri* life cycle.

Many of the known secreted protein encoding genes that were upregulated in the biotrophic phase encode for putative glycoside hydrolases (GH) that act on oligosaccharides making up the plant cell wall. Of the 34 differentially expressed secreted protein genes associated with the biotrophic phase, 11 were glycoside hydrolases from six families of hydrolases, which enables *M. roreri* to interact with the different biochemical components of plant cell walls. This diverse array of polysaccharide degrading enzymes could function together with other hemicelluloses-degrading enzymes, such as those that hydrolyze xylan. All of these polysaccharide degrading enzymes appear to work in concert to loosen the connections between the plant cells, releasing nutrients that can be utilized by the fungus. Pectin is another major component of the intercellular matrix of the cacao pod husk [36]. *M. perniciosa*, the causal agent of WBD on cacao, has been observed to express genes for pectin lyase and pectin methylesterases during infection of parthenocarpic pods [18]. In FPR, pectin is also under attack during the biotrophic phase of *M. roreri.* Genes encoding pectin degrading enzymes are upregulated in this phase.

The biotrophic phase of *M. roreri* is a complex stage requiring controlled cell wall degradation and modification, allowing the invasion of the pod while the fungus remains substantially undetected by plant defenses. All of the *M. roreri* genes encoding plant cell wall enzymes that are induced in the biotrophic phase are able to attack the matrix of the intercellular space. This interaction likely facilitates penetration and spread of the fungus between cells without eliciting major responses from the plant.

In contrast, the expression pattern of genes encoding secreted proteins during the necrotrophic phase of *M. roreri* suggest a more aggressive breakdown of plant structures and the upregulation of aryl-alcohol oxidases, laccases, and a glyoxal oxidase gene during the necrotrophic phase suggests that *M. roreri*, targets lignin for breakdown during the necrotrophic phase. This evidence is further supported by the fact that three of the four aryl alcohol oxidases and three of the four laccases genes are exclusively expressed during the necrotrophic phase (Table 9) [28-30]. There are other upregulated enzymes that may also have a role in these ligninolytic reactions such as a fungal peroxidase, a copper amine oxidase and two dioxygenases. Fungal peroxidases were induced in *M. perniciosa* under nutrient limited conditions [18], while intradiol dioxygenase was shown to be induced in *M. roreri* during necrotrophy in a prior study [21].

Similar to the biotrophic phase, eight glycoside hydrolase (GH) families were also induced in the necrotrophic phase. Although the specific details concerning the exact function of these GH families of genes are lacking, it is interesting to note the differential expression patterns between the biotrophic and necrotrophic phases. GH families shared with the biotrophic phase include families 5, 16 and 18. GH families 28, 43 and 92 are unique to the biotrophic phase, whereas families 3, 9, 10, 35, and 61 are unique to the necrotrophic phase. The GH families unique to the necrotrophic phase include enzymes involved in the degradation of plant cell walls into compounds that the fungus can consume. Complex stage-specific regulation of genes targeting plant cell walls in association with the biotroph/necrotroph shift was also observed in hemibiotrophic *Colletotrichum* species [37]. The differential expression of genes with seemingly related functions provides the opportunity to fine tune aspects of growth and development to the changing conditions and requirements of the two phases of FPR.

Other cell surface-modifying enzymes upregulated in the necrotrophic phase are triacylglycerol lipase precursors and a cutinase suggesting lipid breakdown and modification of the protective surfaces of the plant cells. A necrosis inducing-like protein (NLP) that could be responsible for cell death and nutrient leakage was also found to be up-regulated [38]. NLPs have been identified in many pathogenic associations such as WBD and other biotrophic interactions [39-41]. Two genes from another class of virulence genes, biotrophy-associated secreted proteins BAS (MrBAS1 and MrBAS2) are highly expressed in the *M. roreri* necrotrophic phase. These effector proteins have been shown to accumulate in the biotrophic interface region for the ascomycete *Magnaporthe grisea*[42]; however, their roles in the necrotrophic phase of this interaction are yet to be determined. At the same time in the necrotrophic phase, multiple extracellular peptidases are being upregulated possibly in response to the proteins and peptides that are now available from the necrotic plant cells [43]. Specific activities of *M. roreri* associated with the necrotrophic phase appear to target cell wall and membrane breakdown in associations with enzymes that are linked with the switch of this fungus from biotrophic to necrotrophic phase. The differentially expressed secretome of the necrotroph suggests conditions for rapid growth of the fungus, where plant lignocellulosic cell walls are being degraded and multiple plant components are being released and are available to be used by the fungus as nutrients. The need for stealth has been removed and nutrient acquisition is predominantly to support the rapid growth associated with the necrotrophic phase [21].

### Secretome targeting modification of the fungal cell wall

There is no evidence that haustoria or other cell wall-penetrating structures are formed during the biotrophic phase of *M. roreri*. The slow-growing *M. roreri* mycelia of the biotrophic phase are large pleomorphic cells that fill the spaces between the plant cells [21]. This abnormal mycelial growth habit does have some similarity to haustoria-like structures, although they are outside the cell wall and, possibly provide the fungus with more surface area for cell-to-cell interactions and exchange of metabolites. The necrotrophic mycelia are profuse and grow rapidly in necrotic tissues [21]. These mycelia are thin and elongated. The sporophores and resulting spores represent a second fungal morphology associated with the necrotrophic phase. Genes encoding proteins that are functional in and on the fungal cell wall were differentially expressed between the phases of FPR. Often unique members of the same gene family were differentially expressed.

#### Chitin synthesis and modification

There were five differentially expressed family 18 glycoside hydrolases associated with the biotrophic phase and one associated with the necrotrophic phase. These chitinolytic enzymes could be modifying the chitin structure and the surface of the mycelia. These modifications could protect the mycelia from plant defenses such as plant chitinases [44], thus allowing the fungus to grow intercellularly. Several chitinases are highly expressed in cacao pod tissues throughout development, and can be hyper-induced in response to *M. roreri* infection [21]. Another possibility is that the chitinases attack chitin oligosaccharides released from the fungus, after being attacked by plant chitinases. Additional extracellular chitinases could help eliminate these chitin oligosaccharides and prevent the elicitation of plant defense responses [45].

A chitin deacetylase and a chitin synthase were highly upregulated in the necrotrophic phase. The induction of chitin synthase is consistent with the rapid growth of the fungus initiated during the necrotrophic phase. The chitin deacetylase and related enzymes like exo-beta-glucanases and family 4 carbohydrate esterases can modify chitin, either loosening the chitin polymer bonds or converting the chitin to chitosan. Like the rust fungus *Uromyces viciae-faba*[46]*, M. roreri* may use chitin deacetylase and other enzymes to modify its own cell wall reducing the effectiveness of the plant enzymes. A resistant wall structure may be particularly important in the harsh environment created as plant tissues die. Although cacao defense genes were induced in the later stages of the biotrophic phase of FPR, their induction levels continued to increase once the necrotrophic phase has been initiated [21].

#### PR-1 related proteins

**Figure 3:**
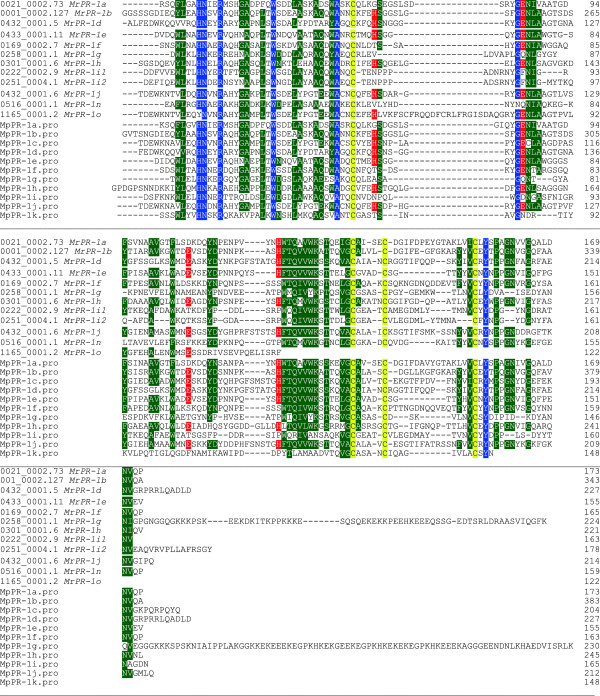
**PR-1 Alignment of the conserved SCP/TAPS protein domains.** Similarities are shown for the PR-1 like-proteins from *Moniliophthora roreri* and *Moniliophthora perniciosa*. Most of the sequences similarities are within the SCP/TAPS domain region. Conserved amino acids are highlighted with blue representing 100% identity and green representing at least a 60% identity. The putative active sites in these proteins are highlighted in red and the conserved cysteines are highlighted in yellow.

Pathogenesis–related 1 proteins (PR-1) are a class of plant genes induced by pathogens during the infection stage and they are partially responsible for systemic acquired resistance responses in those plants. It is of particular interest that plant-like defense proteins would be found in a pathogen and within two species of the same fungal genus. Of the 12 PR-1 related genes identified in the *M. roreri* genome, 10 have homology to PR-1 genes recently described in *Moniliophthora perniciosa*[47]*.* Three of these PR-1-like proteins are upregulated during the biotrophic phase, five are constitutively expressed and only *MrPr-1d* was upregulated in the necrotrophic phase. All of the MrPR-1-like proteins have the SCP/TAPS-like conserved domains (Figure 3). The *MrPR-1 g* and *MrPR-1a* match the expression of their homologs in the biotrophic phase of *M. perniciosa*[47]. Also of interest is the C-terminal extension of the *MrPR-1g* protein, which is found in both *M. perniciosa* and *M. roreri* and could have a specific protein–protein mediated function as purposed by Teixeira *et al.*, [47]. The *MrPR-1b* homolog was constitutively expressed in *M. roreri*, which differs from the case of *M. perniciosa* where it was upregulated in the necrotrophic phase. While the exact function of these PR-1 proteins in fungi is unknown, these fungal-derived PR-1 like proteins may be masking the fungal cell wall from plant defense mechanisms, or they may bind to them preventing their activity. Riviere *et al*., [48] demonstrated that *PR-1a* proteins in tobacco plants regulate the extracellular enzyme activity of β-(1-3)-glucanase activity and plants with PR-1 silenced genes had higher enzyme levels along with a decrease in the deposition of β-(1-3) glucans and callose. Callose deposition in the cell-wall sheath around the plasmodesmata openings is a dynamic regulator mechanism that appears to alter the size of the plasmodesmata orifice, which in turn can modify cell to cell transport by restricting the size of transport molecules [49]. Previous work has shown that *M. roreri* infected cacao pods at 30 DPI have a 1.6 to 2 fold reduction in glucose, phenylalanine and asparagine levels [21], which suggests the fungus has access to these plant metabolites, despite being limited to the intercellular spaces. During the necrotrophic phase (60 DPI) most pod metabolites are severely depleted. The *M. roreri* mycelia, which grow between plant cells, may have access to the plasmodesmata passages and the nutrients that pass through them. The PR-1 like proteins appear to be important to virulence, and it has been suggested that they support systemic spread of the fungus, possibly by limiting susceptibility to host defenses, or they may act as effectors suppressing host defenses [50]. The phase specific expression of the different PR-1 encoding genes is interesting, particularly because of the difference in the host interactions of the biotroph and necrotroph. The PR-1 proteins expressed by *M. roreri* in the different phases could be acting to alter the plant cell walls or as competitive inhibitors of plant PR-1 proteins, thus minimizing the plant defense response, or they could have antimicrobial activity, which would function to prevent subsequent infection and development of competing microorganisms.

#### Hydrophobins

Of the 41 hydrophobins found in the genome, three members of the hydrophobin gene family are up-regulated in the biotrophic phase and 10 members are up regulated in the necrotrophic phase. The hydrophobins interact at the fungal cell surface creating a hydrophobic surface layer and are important in many morphogenetic processes such as sporulation, fruiting body formation and infection structures [51]. Hydrophobins are grouped into three classes Ia, Ib, and II, with Ia and II occurring in ascomycetes and Ib in basidiomycetes [52]. These proteins have the unique ability to self assemble once secreted and they function at the fungal wall-air interface, limiting desiccation and providing protection against both chemical and enzymatic attack [53]. It is easy to postulate the need for different hydrophobins between the two phases of FPR, considering the need to limit detection in the biotrophic phase and the need to protect the surface from enzymatic and chemical attack in the necrotrophic phase. In animal systems at least, hydrophobins are known to prevent host recognition of pathogen-associated molecular patterns, thus preventing the activation of host immune response [54]. Hydrophobins are also of interest because of their potential uses in industrial processes [52].

#### Cerato-platanins and related proteins

Three cerato-platanin (CP) genes are up-regulated in the biotroph and four are upregulated in the necrotroph. Cerato-platanin domain-containing proteins such as EPL (eliciting plant response-like), Snodprot, swollenins and expansins have a double ψβ-barrel fold and a carbohydrate binding site that have been shown to induce plant defense response [55,56]. Cerato-platanins can cause nutrient leakage and cell death in cacao [57,58]. Ectopic expression of the *Magnaporthe grisea* CP gene, *MgSM1*, upregulates the expression of plant defense genes such as PR-1, PR-5 and PDF1.2 and induces local hyper-sensitivity reactions (HR) [59]. This class of fungal proteins is called elicitors or effector proteins. A fungal effector protein found in *Colletotrichum truncatum* (CtCP1) is expressed during the infection stage [60]. Unlike the MgMS1 protein, CtCP1 does not cause HR or elicit a plant defense response, [60] demonstrating that the activity of cerato-platanins can be both host and protein specific. An EPL gene from *Trichoderma* species produces a protein that binds to chitin and self assembles at the air/water interface like a hydrophobin [61]. When MSP1, a snodprot1 homolog protein, was knocked out in *Magnaporthe oryzae,* the fungus was unable to grow within the plant after normal infection suggesting this protein has a key function during the intercellular growth of this fungus [62]. Other CP-family proteins such as snodprot1 homologs are expressed in ecotomycorrhizal fungi *Pisolithus microcarpus* and *Laccaria bicolor*[63], suggesting a role for this family of genes during the plant fungal interaction.

Two expansins were upregulated in the necrotrophic phase of this study. Expansins were first discovered in plants, due to their role in developmental processes involving cell wall modifications, including cell enlargement, by “loosening” plant cell walls [64]. A functional expansin family protein was reported in the basidiomycete *Bjerkandera adusta*[65], and expansin-like proteins have been reported in other fungi, including plant pathogens. Brotman *et al*. [66] reported swollenin, an expansin family protein, in *Trichoderma* that is involved in colonization of plant roots and apparently elicits plant-defense responses. The expansin-like and cerato-platanin proteins may be involved in invasion and pathogenesis of plant tissues by phytopathogens that employ penetration of host/susceptible tissues. Since members of the cerato-platanin family are highly expressed in both phases of FPR, it further supports scenarios in which the fungus uses unique members of complex gene families to facilitate growth and spreading under the conditions unique to each phase.

Another indicator of the biotrophic/necrotrophic switch is the upregulation of several genes in the necrotrophic phase that are associated with fungal reproduction such as the heterokaryon incompatibility protein, a fruiting body-specific gene and a riboflavin aldehyde-forming enzyme. The reproductive stage of the fungus occurs after the necrotrophic phase and is a nutrient intensive process [13]. Cytochrome P450s and hydrophobins have been previously found in necrotic cacao pods infected with FPR [21] and have been shown to be expressed during fungal reproduction, specifically in the fruiting body development [53,67]. However, unlike most basidiomycetes, *M. roreri* has lost the ability to form a basdiocarp or mushroom and sporulates directly from the mycelial mat that it forms on the diseased pod [13].

## Conclusion

The *M. roreri* genome and expression analyses provide insight into the molecular mechanisms of the biotrophic and necrotrophic phases of this important hemibiotrophic pathogen. RNA-Seq analysis revealed differential gene expression during the biotrophic and necrotrophic phases of *M. roreri* during the development of FPR disease of cacao. Focusing on the secreted proteins found in this genome, we observed specific secreted proteins putatively involved in plant cell wall degradation in the biotrophic phase, which seem to degrade and utilize the intercellular spaces. At the same time, they appear to be masking or modifying the fungal cell surface to avoid plant defenses, allowing the fungus to colonize the pod. In contrast, other secreted proteins upregulated in the necrotrophic phase suggest that the fungus continues to protect its mycelia from plant defenses, while actively releasing enzymes and toxins that have been reported to attack the plant cell wall components and utilizing plant nutrients that are released during plant cell death. These findings support the existing pathogenesis model for FPR while giving greater details regarding the identity of putative secreted proteins involved in the different stages, and information regarding the differential use of unique members of complex gene families. Due to the high number of genes that encode hypothetical proteins in this genome and that are expressed during the disease process more research is needed to fully understand the various phases of this hemibiotrophic fungus.

## Methods

### Biological material, libraries construction and sequencing

The *M. roreri* clone (MCA2977) was isolated from the state of Los Rios in Ecuador. It was grown and maintained on a yeast extract (5 gL^−1^) and glycerol (20 mL L^−1^) medium. Cultures were incubated at 27°C at 200 rpm for 7 d. Total DNA was extracted using a cetyl trimethylammonium bromide (CTAB) method [68].

### Genome assembly

*Moniliophthora roreri* DNA sequences were obtained by a hybrid sequencing approach using GS FLX and Illumina sequencing, and the whole-genome shotgun strategy. The GS FLX sequencer (454 Life Sciences/Roche) was used to produce 3,440,399 single-end reads with a mean length of 230 bp and 417,891 reads from paired end runs with 2Kbp insert size. An Illumina Genome Analyzer was used to produce 30,826,974 read pairs (2 × 76 bp, insert size of 365 bp). The Illumina data was filtered to remove reads from mitochondrial genome and plasmids. Illumina data was error-corrected using Quake v0.3 with default parameters. In total, 24,405,509 read pairs passed Quake error correction. The final hybrid assembly of both 454 data and Illuminia data was performed by using the Newbler v.2.6 assembler (default parameters with overlap parameter > = 40 and identity to > =95%, http://www.454.com/products/analysis-software/) [69,70].

### Combined gene models

#### Ab-initio gene models prediction

Genefinding prediction programs, Genemark-ES v2.3 [71] and Augustus v2.3.1 [72,73] programs optimized with the Exonerate v2.2.0 protein2genome model [74] were used to perform the *ab-initio* gene prediction. Genefinding prediction program Genemark.hmm [75] was executed in self-training mode and ORFs larger than 200 bp were retained. The Genemak-ES predicted proteins were aligned against the GenBank NR, using BLASTP with a e-value cutoff of 1e-10. Finally, EVidence Modeller (EVM) [76] was used to combine the predictions from Genemark-ES, Augustus and Exonerate spliced protein alignments and generate a consensus gene prediction. Select gene models were manually inspected, especially incomplete genes 5' and 3' ends of the scaffold.

### Automatic annotation

The automatic annotation program AutoFACT [77] was used for functional annotation of gene models. The main contribution of AutoFACT is the capacity to conduct the annotation based on sequence similarity searches in several databases. For this, we used the BLASTp (e-value cutoff of 1e-5) to align the gene models against various proteins databases: non-redundant (NR) database at NCBI, SwissProt–databases containing only manually curated proteins [78], uniref90 and uniref100–databases containing clustered sets of proteins from UniProt, Pfam–database of proteins families [79] and KEGG–database of metabolic pathways [80]. Open reading frames were also annotated using Blast2GO (http://www.blast2go.com/b2ghome) [81].

### Transposable elements

The *M. roreri* and *M. perniciosa* genomes were scanned for transposable elements. Each genome was fragmented *in silico* into 1000-bp fragments. These fragments were compared to a database of full length LTR retroptransposons developed by the program LTR struc [82] of LTRs associated with *M. roreri* and *M. perniciosa* using the BLAST program. Fragments with significant E-values (E-04) and better were sequestered. The rest of the fragments with no hits to LTR retrotransposons were scanned against the repbase database [83], which is a collection of sequences representing repetitive DNA from different eukaryotic species using the Blast program.

The *M. roreri* and *M. perniciosa* genomes were also scanned for repeptive DNA using the program Repeat Masker with full Repbase. However, this scan did not find all the LTR and repetitive elements. Thus, repeat Modeler was also used to find *denovo* repeats and used as the database for input into Repeat Masker. This analysis is shown (Additional file 1 Repeat modeler sheet) *RepeatMasker Open-3.0*. <http://www.repeatmasker.org>.

### Transcriptome

#### Transcriptome analysis in infected pods

Briefly, the pods used in this study were derived from flowers hand pollinated 60 days prior to infection with a spore suspension of 1×10^8^ spores ml^-1^[21]. The plant material used was a segregating F1 population of progeny from highly susceptible clones (PA16 × SIC433 and PA16 × EEG29). Seven infected pods were frozen in liquid nitrogen at 30 days post infection (DPI) and another seven at 60 DPI. Based on the typical progression of FPR symptoms [21], malformed green pods were selected for RNA-Seq and RT-qPCR from 30 DPI pods which represents the biotrophic phase. Necrotic sporulating pods were selected for RNA-Seq and RT-qPCR from 60 DPI pods, which represents the necrotrophic phase.

### RNA extraction

Each infected pod was broken up and coarsely ground under liquid nitrogen and approximately 1 cm^3^ was used for processing. The pod material was ground finely and transferred to a disposable 50 mL centrifuge tube containing 15 mL of 65°C extraction buffer [84]. Additional extraction methods were conducted as in Melnick *et al*. [18]. The cDNA was synthesized using the Invitrogen (Carlsbad, CA) Superscript VILO kit, following the manufacturer’s directions.

### RNA sequencing

For the genome-wide analyses of expression patterns, cDNA was generated using a routine RNA library preparation TruSeq protocol developed by Illumina Technologies (San Deigo, CA). Using the kit, mRNA was first isolated from total RNA by performing polyA selection step, followed by construction of single end sequencing libraries with an insert size of 160 bp. Single-end sequencing was performed on six samples (three 30 DPI and three 60 DPI) using the Illumina HiSeq platform. Samples were multiplexed with unique six-mer barcodes generating 404,820,524 filtered (for Illumina adapters/primers, and PhiX contamination) 1×50 bp paired end reads.

### Expression analysis

Reads from 30 DPI and 60 DPI libraries were mapped to the nucleotide sequences of predicted protein coding genes of the *M. roreri* genome using the short read aligner Bowtie-0.12.7 which utilizes a Burrows-Wheeler index [85]. Count data were obtained for each coding sequence. Estimation and statistical analysis of expression levels using count data of each gene with 3 replicates for each library was performed using the DEseq package [86] and R x64 2.15.2 program. (http://www.r-project.org/).

After RNA-Seq, twenty five genes were chosen for analysis by RT-qPCR across the full set of infected pods. For RT-qPCR analysis, seven replicate malformed green pods 30 DPI and seven replicate necrotic sporulating pods (60 DPI) were used. RT-qPCR analysis was conducted following Bailey *et al*. [21], using Brilliant III® SYBR® Green Q-PCR Master Mix (Agilent, Santa Clara, CA). Primer sources, sequences for the *M. roreri* genes are in Additional file 3. *M. roreri* ESTs were chosen based upon results of RNA-Seq analysis. RT-qPCR was conducted to determine the changes in expression of *M. roreri* genes between the biotrophic and necrotrophic phases of FPR. The ddCt method was used to calculate the fold-change between the 30 and 60 day DPI samples [87,88].

### Statistical analysis of data

A two-way ANOVA of the RNA-Seq data was conducted using the general linear model (PROC GLM) followed by Tukey post-hoc testing (α = 0.05) using SAS 9.3 (SAS Institute Inc., Raleigh, NC, USA) to analyze pod data. For RT-qPCR, relative transcript levels were determined following Pfaffl [89].

### Secreted proteins

17,920 protein coding sequences were scanned for signal peptides using signalP [90,91]. The resulting proteins containing signal peptides were scanned for transmembrane proteins using the TMHMM program (Prediction of transmembrane helices in proteins) [92]. Proteins with no more than two transmembrane domains were sequestered for further analysis. The resulting proteins were scanned for GPI-anchored proteins [93,94] using FragAnchor, which is based on the tandem use of a Neural Network predictor and a Hidden Markov Model predictor (http://navet.ics.hawaii.edu/~fraganchor/NNHMM/NNHMM.html) [95]. Proteins that were highly probably and probable of having a GPI-anchor were discarded. Thus, using bioinformatics, proteins with signal peptides, with two or less transmembrane domains and without GPI-anchors were considered for possible secretion.

### Venn diagram

Using a bi-directional blast method the *M. roreri*, *M. perniciosa*, *Laccaria bicolor*, *Coprinus cinereus,* and *Ustilago maydis* genomes were compared and used to generate a 5-way Venn diagram. In addition, five tax plots for all five fungal species were created using each genome as a reference, respectively. Protein sequences that share an expected E-value of E-04 were considered matches.

### Dot plots

Dot plots between the large contigs (50,000 bp or larger) of *M. roreri* and *M. perniciosa* were created using the whole genome aligner MUMmer3.22. The NUCmer and MUMmerplot programs from the MUMmer suite were used [96,97].

## Availablity of supporting data

This *M. roreri* Whole Genome Shotgun project has been deposited at DDBJ/EMBL/GenBank under the accession AWSO00000000. The version described in this paper is version AWSO01000000. The sequencing data used in this study can be found under NCBI BioProjects. BioProject PRJNA213737 is linked to the genome data and PRJNA229176 was established for the transcriptome data.

## Abbreviations

*M. roreri*: *Moniliophthora roreri*; *M. perniciosa*: *Moniliophthora perniciosa*; FPR: Frosty Pod Rot; CDS: Coding sequences; OFRs: Open reading frames; RNA-seq: RNA sequencing; WBD: Witches’ broom disease; Mbp: Million base pairs; NLP: Necrosis inducing-like protein; NP-50: Statistical measure of average length of contigs in a Draft sequence.

## Competing interests

The authors declare that they have no competing interests.

## Author’s contributions

LWM conceived the study, participated in its design and coordination, ARS lead and drafted the manuscript. GGLC participated in the sequence alignment and bioinformatic analysis of the genome. DPTT sequence analysis and helped to draft the manuscript. PJPLT sequence analysis and helped to draft the manuscript. MFC participated in the sequence alignment and bioinformatic analysis of the genome. SCS conducted 454 sequencing and participated in the sequence alignment. JEC conducted 454 sequencing and participated in the sequence alignment. MJG participated in its design and coordination of the study, PSU lead. PM conducted Illumina sequencing and participated in the sequence alignment. AF conducted RNA sequencing and bioinformatics analysis. TR conducted RNA sequencing and bioinformatics analysis. JC conducted field studies, collected samples and participated in the statistical analysis of data. RED sequence analysis and helped to draft the manuscript. JS participated in the sequence alignment, bioinformatic analysis of the RNA-seq data and helped to draft the manuscript. RLM carried out molecular genetic studies, participated in the statistical analysis of data and helped to drafted the manuscript. GAGP participated in its design and coordination of the study, Brazilian lead. BAB conceived the study, participated in its design and coordination, carried out molecular genetic studies and helped to draft the manuscript. All authors read and approved the final manuscript.

## Supplementary Material

Additional file 1 **This file contains the Repeat Modeler file and a complete list of the Venn diagram intersects; A, AB, ABC and ABE discussed in the text.** There is a sheet for each set and intersect and RNA-Seq data are provided for each gene in that intersect. Click here for file

Additional file 2 **This file contains the complete list of the 1535 secreted genes and the 125 GPI-anchor containing proteins identified in the *M. roreri *** **genome.** The file contains RNA-Seq data for each gene and RT-qPCR data for a number of selected genes. The corresponding *M. perniciosa* homolog is also listed with the homology E-value. Click here for file

Additional file 3 **This file contains the complete set of RT-qPCR primers used in this study.** The file provides the primer name, nucleotide sequence, *M. roreri* gene Id, PCR fragment size and the nucleotide sequence for each fragment. Click here for file
